# Evaluation of the Recognition of Stroke in the Emergency Room (ROSIER) Scale in Chinese Patients in Hong Kong

**DOI:** 10.1371/journal.pone.0109762

**Published:** 2014-10-24

**Authors:** Hui-lin Jiang, Cangel Pui-yee Chan, Yuk-ki Leung, Yun-mei Li, Colin A. Graham, Timothy H. Rainer

**Affiliations:** 1 Accident and Emergency Medicine Academic Unit, The Chinese University of Hong Kong, Hong Kong, China; 2 Emergency Department, The Second Affiliated Hospital of Guangzhou Medical University, Guangzhou, China; University of Washington, United States of America

## Abstract

**Background and Purpose:**

The objective of this study was to determine the performance of the Recognition Of Stroke In the Emergency Room (ROSIER) scale in risk-stratifying Chinese patients with suspected stroke in Hong Kong.

**Methods:**

This was a prospective cohort study in an urban academic emergency department (ED) over a 7-month period. Patients over 18 years of age with suspected stroke were recruited between June 2011 and December 2011. ROSIER scale assessment was performed in the ED triage area. Logistic regression analysis was used to estimate the impacts of diagnostic variables, including ROSIER scale, past history and ED characteristics.

**Findings:**

715 suspected stroke patients were recruited for assessment, of whom 371 (52%) had acute cerebrovascular disease (302 ischaemic strokes, 24 transient ischaemic attacks (TIA), 45 intracerebral haemorrhages), and 344 (48%) had other illnesses i.e. stroke mimics. Common stroke mimics were spinal neuropathy, dementia, labyrinthitis and sepsis. The suggested cut-off score of>0 for the ROSIER scale for stroke diagnosis gave a sensitivity of 87% (95%CI 83–90), a specificity of 41% (95%CI 36–47), a positive predictive value of 62% (95%CI 57–66), and a negative predictive value of 75% (95%CI 68–81), and the AUC was 0.723. The overall accuracy at cut off>0 was 65% i.e. (323+141)/715.

**Interpretation:**

The ROSIER scale was not as effective at differentiating acute stroke from stroke mimics in Chinese patients in Hong Kong as it was in the original studies, primarily due to a much lower specificity. If the ROSIER scale is to be clinically useful in Chinese suspected stroke patients, it requires further refinement.

## Introduction

Stroke is a major cause of death and the principal global cause of adult disability. In China, stroke is the second commonest cause of death [1]. The number of patients who die from stroke is three times more than that from coronary heart disease [2]. The benefits of emergency medical services (EMS) activation by patients with stroke symptoms appear to occur in both the prehospital and in-hospital settings. For faster access to acute stroke management, stroke patients need to be accurately identified in the emergency department (ED), and ideally prior to ED arrival. Prehospital staff (e.g. emergency paramedics) are the first medical contact in 38–70% [3]–[6] of stroke patients. However, studies have demonstrated that paramedics have inadequate levels of stroke knowledge [7] and identify only 61–72% [8]–[10] of stroke patients. Many studies from western countries have validated the accuracy of various scoring systems to determine suspected stroke in the prehospital setting and ED, such as the the Los Angeles prehospital stroke screen (LAPSS score), Cincinnati prehospital stroke scale, Face Arm Speech Test (FAST), the Melbourne Ambulance Stroke Screen (MASS score) and Recognition Of Stroke In the Emergency Room (ROSIER) scale [11]–[15].

The ROSIER scale, which was developed in a UK population, has been designed to provide physicians in the emergency department with a framework which can be used to assess patients with suspected stroke, to facilitate early identification of acute stroke and appropriate referral. The ROSIER scale uses rapidly assessable elements of history and physical examination to produce a score between −2 and +6. The original study recommended that a score>0 indicates a likely stroke, and reported a sensitivity of 92–93%, a specificity of 83–86%, a positive predictive value (PPV) of 88–90%, and a negative predictive value (NPV) of 88–91%. External validation of diagnostic scores in different clinical cohorts is important. Two previous studies attempted to validate the score in Chinese patients; but one was done in the pre-hospital setting, and the second used patients both from a neurology unit and an emergency room [16]–[17]. The population in Hong Kong (HK) is mainly Chinese, but their lifestyle is becoming increasingly westernized. Validation of the ROSIER scale is an important step before the score can be safely used to identify Chinese patients with stroke. The aim of this study was to investigate whether the ROSIER scale was a valid tool for identifying stroke in Chinese patients with suspected stroke presenting to an ED in Hong Kong.

## Methods

### Study design and study setting

In this prospective cohort study, conducted from 1 June 2011 to 31 December 2011, all patients with suspected stroke or transient ischaemic attack (TIA) were included. The study was approved by the Institutional Review Board of the Chinese University of Hong Kong and written consent was obtained from all patients or the closest available relatives.

This study was conducted in the ED of the Prince of Wales Hospital (PWH), a tertiary referral centre affiliated with the Chinese University of Hong Kong. PWH is a university hospital with 1400 beds, which is located in the New Territories in Hong Kong. The ED sees more than 150 000 new ED patients per annum, serves a local population of approximately 800 000 people, and is the regional neurosciences centre for around 1.5 million people.

### Inclusion and exclusion criteria

Consecutive patients ≥18 years old, presenting to the ED with symptoms or signs suggestive of stroke or TIA were included in the study. The following patients were excluded: traumatic brain injury with an external cause such as motor vehicle crashes and falls; incomplete medical records; patients that did not present first to the ED (e.g. direct admission to a ward); and in accordance with the criteria for the original ROSIER scale, patients with subarachnoid haemorrhage (SAH), subdural haematoma (SDH) and TIA without symptoms and signs during this period. The inclusion and exclusion criteria were the same as the original article [15] thus allowing meaningful comparison.

### Definitions

Stroke was defined as a focal or global neurological deficit with symptoms lasting for 24 hours, or resulting in death within 24 hours, which after investigation was thought to be due to a vascular cause. TIAs were defined as clinical syndromes characterized by an acute loss of focal cerebral or monocular function with symptoms lasting less than 24 hours and thought to be caused by inadequate blood supply as a result of thrombosis or embolism.

Stroke mimics were defined as manifestations of nonvascular disease processes when a stroke-like clinical picture is produced. The presentation resembles or may even be indistinguishable from ischaemic stroke [18].

Somatization disorder causes patients to suffer from neurological symptoms, such as numbness, blindness, paralysis, or fits without a definable organic cause. It is thought that symptoms arise in response to stressful situations affecting a patient's mental health [19].

### Study protocol

All patients referred to the ED with suspected stroke were examined by research staff who completed the ROSIER proforma and data collection. The research staff were fully trained, specialist stroke nurses or a consultant in emergency medicine who performed the initial clinical examination. In addition to the ROSIER assessment or where assessment was difficult, the default was to use the NIHSS score (see Appendix S1).

Data collected prospectively during this study included patient characteristics, stroke referrals, onset and admission times, assessment time, clinical symptoms and signs, risk factor profile, Glasgow Coma Score (GCS) score, National Institutes of Health Stroke Scale (NIHSS), non-invasive blood pressure, imaging findings, final diagnosis, and early outcome at discharge. All patients suspected of stroke were reviewed by the stroke team which included four stroke nurses and two specialist doctors. The final diagnoses were made after their assessment and after review of clinical symptoms and the acute neuro-imaging (computed tomography [CT] and magnetic resonance imaging [MRI]), and this was used as the reference standard for diagnosis in the study.

### Statistical analysis

Data was entered into a Microsoft Access database, and statistical analyses were done using SPSS version 17 (SPSS Inc., IBM Corporation, Chicago, IL) and MedCalc®version 11.5.1 (Mariakerke, Belgium). The prevalence of symptoms and signs were calculated. Baseline clinical characteristics were compared using Chi-square tests, unpaired t tests, and the Mann-Whitney U test. To obtain the ROSIER scale cut off points for discriminating between patients with stroke and stroke mimic, we constructed receiver operating characteristic (ROC) curves and calculated the area under the ROC curve (AUC) with 95% confidence intervals (CI). A P value of <0.05 was considered statistically significant. The sensitivity, specificity, PPV, and NPV of the ROSIER scale were calculated. Our primary aim was to evaluate in Chinese patients the suggested cut off of>0 as suggested from the original ROSIER scale. Differences between stroke patients and stroke mimic patients were assessed using descriptive statistics and standard tests of significance (as indicated). Univariate logistic regression analyses were initially performed on all ROSIER variables with p<0.1 for patients with either stroke or stroke mimic. Subsequently all ROSIER variables were analysed using multivariate logistic regression.

## Results

A total of 766 patients with suspected stroke and TIA were initially recruited to the study. Fifty-one patients were excluded (four patients had incomplete medical records; two cases were not assessable; 45 cases did not meet the original ROSIER scale criteria (patients with SAH, SDH and TIA were excluded). This left 715 patients for analysis. Of these, 371 (52%) had symptoms owing to a final diagnosis of probable or definite acute cerebrovascular disease, and 344 (48%) had symptoms owing to other illnesses. Of these 715 patients, 420 patients had an onset time of less than 24 hours, and 295 patients had an onset time longer than 24 hours prior to assessment; 713 patients underwent neuroimaging: 712 patients had a CT scan, and 225 patients had an MRI scan.

Table 1 shows the baseline characteristics and ROSIER scales of these patients. The stroke patients were older than stroke mimic patients [72 years (SD13) vs 69 years (SD14), P = 0.003]. Male sex (58% vs 49%, P = 0.018), past history of hypertension, (70% vs 60%, P = 0.007), history of smoking (17% vs 8%; P<0.001), and history of atrial fibrillation (AF), 16% vs 7%; P<0.001) were more frequent in stroke patients than in stroke mimics. Patients with a past history of previous stroke and somatization were less frequent in stroke patients than in stroke mimics (25% vs 32%, P = 0.049, 1% vs 5%, P<0.001; the first SBP and DBP in stroke patients were higher than in stroke mimics. Stroke patients showed significantly higher ROSIER scales compared to stroke mimics [median 2 (IQR1–3) versus 1 (IQR0–2), P<0.001].

**Table 1:** Basic characteristics and Rosier score of stroke and stroke mimic patients (n = 715).

	Stroke or TIA (n = 371)	Stroke mimic (n = 344)	P Value
Age, mean (SD), years	72 (13)	69 (14)	0.003*
Sex, male, n (%)	214 (58)	168(49)	0.018*
**Presentation**			
Within 3 hours	15(4)	8(2)	0.193
Within 24 hours	168(49)	252(68)	<0.001
**Clinical Symptoms and signs**			
Vertigo	60(17)	57(16)	0.886
Confusion	15(5)	7(2)	0.432
Facial paresis	139(38)	65(19)	0.001*
Arm paresis	240(65)	131(38)	<0.001*
Leg paresis	238(64)	140(41)	<0.001*
Speech disturbance, n(%)	220(59)	89(26)	<0.001*
Visual field defect	70(19)	23(7)	<0.001*
Loss Of Consciousness/syncope, n(%)	34(9.2)	47(13.7)	0.58
Systolic blood pressure (mmHg), mean(SD)	165(30)	150(27)	<0.001*
Diastolic blood pressure (mmHg), mean(SD)	84(20)	79(15)	<0.001*
Pulse (bpm), mean(SD)	78(16)	79 (17)	0.607
GCS, n (%)			
GCS ≥ = 14	328(93)	319(88)	0.123
GCS>8-<14	28(8)	18(5)	
GCS < = 8	15(5)	7(2)	
**Past Medical History, n(%)**			
Hypertension	258(70)	206(60)	0.007*
Smoker	64(17)	27(8)	<0.001
Diabetes mellitus	123(31)	107(33)	0.558
Ischemic heart disease	45(12)	49(14)	0.403
Atrial fibrillation	61(16)	25(7)	<0.001*
Previous stroke	94(25)	110(32)	0.049*
Somatization	2(1)	16(5)	<0.001*
**ROSIER scale, median (IQR)**	2(1–3)	1(0–2)	<0.001*

*Statistically significant.

Table 2 shows that the distribution of stroke aetiologies were ischaemic stroke in 302 (81%), haemorrhagic stroke in 45 (12%), and TIA in 24 (7%). The most common stroke mimics were spinal neuropathy, dementia, labyrinthitis and sepsis which together comprised 33% of the 334 stroke mimic cases.

**Table 2:** Primary diagnosis in study participants (n = 715).

	Stroke or TIA (n = 371)	Stroke mimic (n = 344)
**Stroke classification**		
Ischaemia	302(81)	-
Primary intracerebral haemorrhage	45 (12)	-
Transient ischaemic attack	24(7)	-
**Non-stroke diagnosis**		
Spinal neuropathy	-	34(10)
Dementia	-	27(8)
Labyrinthitis	-	27(8)
Sepsis	-	27(8)
Musculoskeletal disorder	-	24(7)
Syncope	-	24(7)
Hypertension	-	21(6)
Somatisation	-	20(6)
Metabolic disorder	-	18(5)
Uncertain	-	17(5)
Brain and other tumour	-	16(5)
Peripheral Neuropathy	-	16(5)
Encephalopathy	-	14(4)
Numbness	-	14(4)
Transient global amnesia	-	13(4)
Others**	-	32 (9)

**Others included: Parkinson's disease (n = 7); seizure (n = 5); Medication side effect (n = 5); Migraine (n = 5); intracranial venous sinus thrombosis (n = 2); one of each of ataxia, dural, arteriovenous malformation, heat stroke, optic neuritis, postural hypotension, Shy-Drager syndrome, systemic lupus erythematosus (SLE) and syringomyelia.

Table 3 shows the comparison of sensitivity and specificity. In our study, at a cut off>0, the AUC was 0.723.

**Table 3:** Diagnostic performance of ROSIER instruments in Chinese patients and original ROSIER scale paper [15].

	ROSIER Scale at cutoff>0 in this study(95%CI) n = 715*	ROSIER Scale at cutoff>0 in Lancet Paper(95%CI) in development phase n = 343	ROSIER Scale at cutoff>0 in Lancet Paper(95%CI) in validation phase n = 160*
Sensitivity	87(83–90)	92(89–95)	93(89–97)
Specificity	41(36–47)	86(92–90)	83(77–89)
PPV	62(57–66)	88(85–91)	90(85–95)
NPV	75(68–81)	91(88–94)	88(83–93)

*All suspected patients including ischaemic strokes, intracerebral haemorrhages, subarachnoid haemorrhage, subdural haematoma and transient ischaemic attacks with or without symptoms and signs.

Table 4 shows that odds ratios for stroke were increased for asymmetric facial/arm and leg weakness, speech disturbance and visual field defect. In the multivariate analyses, the adjusted odds ratio for stroke was significantly decreased for LOC/syncope, and significantly increased for asymmetric arm weakness, speech disturbance and visual field defect.

**Table 4:** Univariate and multivariate logistic regression analysis of the items of ROSIER scale (n = 715).

	Univariate analysis		Multivariate analysis	
	OR(95%CI)	P Value	OR(95%CI)	P Value
LOC/syncope	0.64(0.399–1.018)	0.059	0.32(1.784–5.859)	<0.001
Seizure activity	1.64(0.474–5.634)	0.436		
Asymmetric facial weakness	2.57(1.827–3.621)	<0.001	1.23(0.832–1.828)	0.298
Asymmetric arm weakness	2.98(2.197–4.039)	<0.001	1.94(1.194–3.154)	0.007
Asymmetric leg weakness	2.61(1.928–3.527)	<0.001	1.22(0.748–1.979)	0.429
Speech disturbance	4.17(3.037–5.737)	<0.001	3.41(2.381–4.889)	<0.001
Visual field defect	3.25(1.975–5.333)	<0.001	2.41(1.332–4.343)	0.04

**Figure 1:**
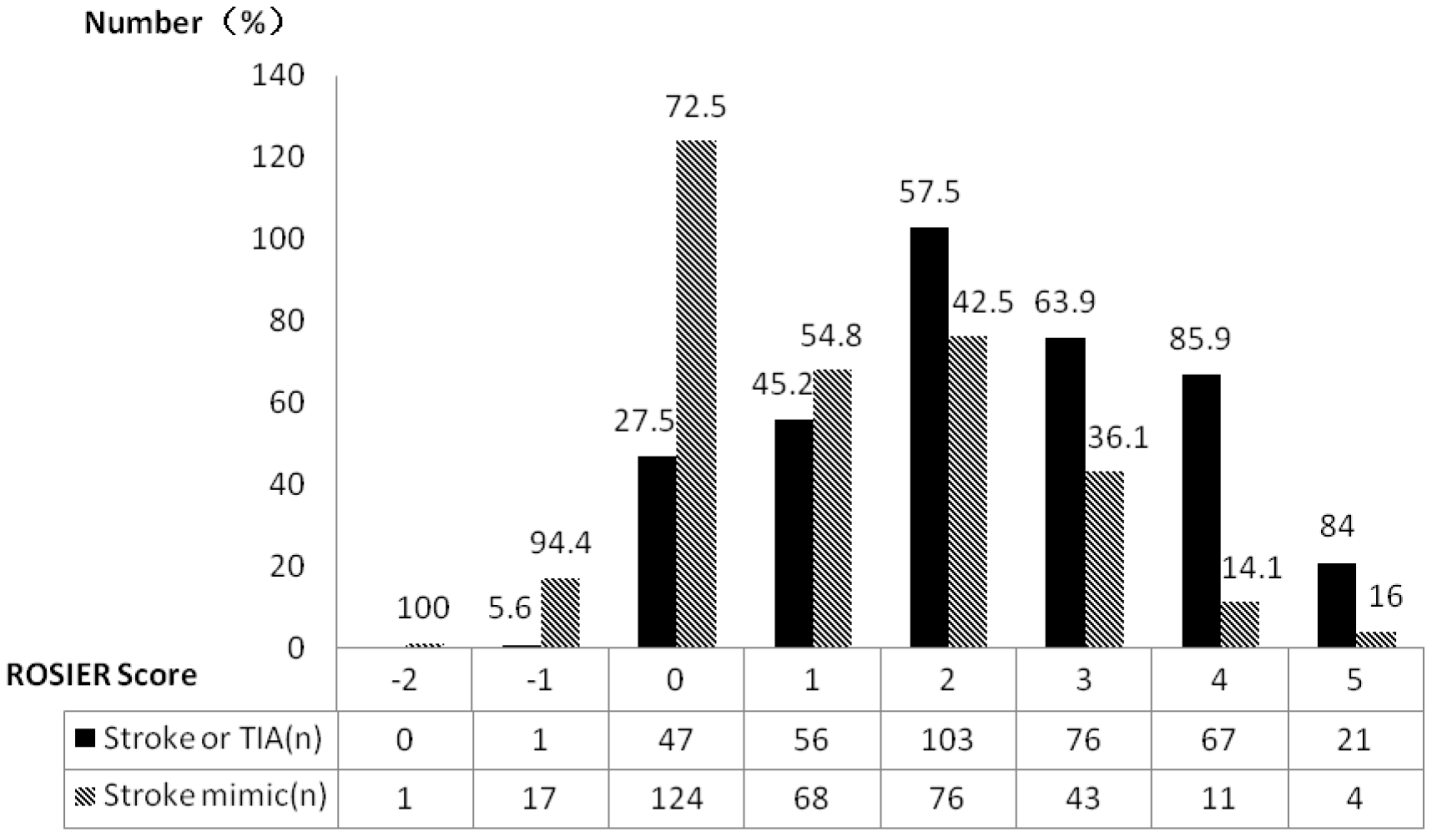
Proportions of stroke diagnosis among different ROSIER scale (n = 715).

Figure 1 shows the distribution of the ROSIER scale categories. At a cut off>0, there are 323 stroke cases, and 202 stroke mimics, and at a cut off ≤0, there are 48 stroke cases, and 141 stroke mimics. The overall accuracy at cut off>0 is (323+141)/715 = 0.65, or 65%.

## Discussion

Formalized stroke assessment tools, based upon easily collected clinical variables, may help emergency department staff in identifying hyperacute stroke more quickly and efficiently. The ROSIER scale is one of the most widely recommended scores in the UK [20]–[22].

Our study is the largest to date to assess the value of the ROSIER scale in a non-Western population. At the suggested cut-off of>0, the ROSIER scale demonstrated lower sensitivity (87%) and specificity (41%) for the identification of Chinese suspected stroke patients compared to the original study [15]. A study reported by Whiteley in United Kingdom (UK) also showed that the sensitivity (83%) and specificity (44%) of the ROSIER scale in their setting was not comparable to that of the original study [23]. Although the sensitivity and specificity in our paper and Whiteley's paper is similar, nevertheless there are major differences in the clinical characteristics of our two settings. Compared with the UK, patients in Hong Kong present later after symptom onset, have different clinical presentations, less atrial fibrillation and different proportions of ROSIER score categories. One Irish study of 50 patients confirmed that the ROSIER score appears to be a sensitive instrument for assisting emergency doctors in identifying stroke patients [24] but the sample size was limited.

In Hong Kong, 95% of the population is Chinese [25] and there are significant differences in the distribution of stroke subtypes between Chinese patients [26], [27] and patients of Western origin. There is a higher proportion of intracerebral haemorrhage in Chinese compared with Western populations [28]–[31]. Also, in our study there were less ischaemic stroke and TIA patients (91% versus 86%), and more haemorrhagic stroke patients (12% versus 8%) than in the validation phase of the original study [15]. The proportion of stroke types in Chinese also varies from 43.7% to 78.9% for cerebral infarction, and from 18.8% to 47.6% for intracerebral haemorrhage [32,–34]. These differences in subtype patterns are postulated to be due to differences in genetic, clinical, environmental and lifestyle factors [27], [35], [36].

There are also differences in the proportion of stroke mimics between the validation phase of the original study (37%) and this study (48%). Seizures (<9%) and syncope (7%) in Hong Kong are less common stroke mimics than in the UK (57%) [15]. Another UK study found that seizures and complex migraines comprised up to 33.9% of stroke mimics [37], whilst in Greece [38] the principal stroke mimics were aphasic disturbances (27.3%) and dizziness or fainting (27.3%).

Any change of the weight of odds ratio of parameters in the ROSIER scale in the study cohort could affect its diagnostic value in Chinese patients. Compared with the original ROSIER scale, three neurological signs in Hong Kong, namely facial weakness (2.57 vs 27.0), arm weakness (2.98 vs 16.6) and leg weakness (2.61 vs 13.1) all had reduced odds ratios for the diagnosis of stroke and stroke mimic. A study investigated the diagnosis value of ROSIER scale in ambulance clinicians showed only 3 items of ROSIER scale, facial weakness (OR:2.433,95%CI:1.368–4.326), arm weakness (OR: 3.593,95%CI: 1.993–6.478), and seizure activity (OR: 3.652,95%CI: 1.279–10.426), significantly predicted stroke final diagnosis [39].

Previous studies have suggested that blood pressure (BP) is a particularly important risk factor for stroke in the Chinese population [40]. The risk of stroke associated with hypertension is consistently and significantly greater in Chinese than in Caucasians [41]. In our study, stroke patients more frequently had a history of hypertension compared to stroke mimic patients. The first blood pressure on ED admission was elevated in 95 patients (64%). The mean systolic BP reached 165 mmHg in Chinese stroke patients compared with 150 mmHg in the UK [15].

However, having a previous stroke can affect the diagnostic accuracy in this study. If patients have any prior neurological deficits, the assessment of the ROSIER score becomes especially complicated. The assessment principle of the ROSIER score is according to the NIHSS Guiding Principles: items are scored only if definitely present. So patients with previous neurological deficits will have higher ROSIER scores by default. The ratio of previous stroke patients both in both the stroke (25%)and stroke mimic (32%) groups in our study are higher than those in Nor's report (18% in both groups). This larger proportion of patients with previous stroke in our study may have affected the observed diagnostic accuracy.

This study had the following strengths. The ethnicity of the patient population is fully Chinese and therefore different from most studies investigating ROSIER. The sample size is large, especially compared with the original paper.[15] The study was conducted in the triage station of the emergency department and by well trained neurology and emergency research staff. The recruitment and analysis is exactly that same as the original paper [15] therefore allowing meaningful comparison. As the ROSIER score involves clinical assessment, no confusion should result in translating from English to Chinese.

There are several limitations in this study. Firstly, this is a single-centre study and may not reflect Asia in general. Stroke prevalence varies according to geography and ethnic background in China and this may limit the generalisability of our results.

Secondly, our original research purpose was to validate the ROSIER score in the emergency room. Some features, such as the past medical history and clinical signs (such as parasthesia and incoordination) which may increase the diagnostic value of stroke were not included in this research.

Thirdly, most recruits in our study are elderly which are a very heterogeneous group wit multiple morbidities.

In conclusion, the ROSIER scale was not as effective at differentiating acute stroke from stroke mimics in Chinese patients in Hong Kong as it was in the original studies, primarily due to a much lower specificity. There is a continuing need to develop and validate appropriate tools in the ED to improve diagnostic accuracy in the detection of stroke in the Chinese population.

## Supporting Information

Appendix S1 **Assessment of patients in coma.**
(DOCX)
