# Supplementary material for: Evaluation of the Recognition of Stroke in the Emergency Room (ROSIER) Scale in Chinese Patients in Hong Kong
Source: PLoS One. 2014 Oct 24;9(10):e109762. doi: 10.1371/journal.pone.0109762 (PMC4208764; doi:10.1371/journal.pone.0109762)
Supplement: Appendix S1 — Assessment of patients in coma. (DOCX) [file pone.0109762.s001.docx]

**Appendix 1: Assessment of patients in coma**

The original ROSIER scale did not give details of how the suspected stroke patients with coma (the definition was GCS<=8) were assessed. We chose to follow the criteria of NIHSS score and the clinical examination rule:

**LOC/syncope:** Following the criteria in the NIHSS score, 1 point was given if the category in NIHSS Item 1b LOC questions and Item 1c LOC commands was >=1.

**Asymmetric facial weakness:** Following the criteria in the NIHSS score, 1 point was given if the category in NIHSS Item 4 awas symmetric facial palsy >=1. The method for checking responsiveness is to stimulate each nasal passage separately using a cotton-tipped applicator.

**Asymmetric arm /leg weakness**: Following the criteria in the NIHSS score, 1 point was given if the category in NIHSS Item 5&6 asymmetric motor arm and leg was >=1. The method of checking when patients are coma is to test tone in the normal by picking up the arm and letting it fall. Both sides are compared for evidence hemiplegia. In coma and acute cerebral hemiplegia, the muscle stretch reflexes may be normal or reduced on the paralysed side.

**Speech disturbance:** Following the criteria in the NIHSS score, 1 point was given if the category in NIHSS Item 9 was best language >=1 and Item 10 was dysarthria>=1).

**Visual field defect:** Following the criteria in the NIHSS score, 1 point was given if the category in NIHSS for Item 2 best Gaze was >=1 and Item 3 visual deficits was >=1.

3.2 The final score of ROSIER scale in coma patients is shown in table 2. There were 22 patients with GCS<=8 in our study. Patients with GCS<=8 showed significantly lower ROSIER scales compared to patients with GCS>8 [median 2 (IQR0-3) versus 3 (IQR2-4)], P=0.001.

Table 1: The comparison of ROSIER scale of Suspected stroke patients with different GCS

|  | Suspected stroke patients with GCS<=8  (n=22) | Suspected stroke patients with GCS>8  (n=693) |  |
| --- | --- | --- | --- |
| ROSIER scale, median (IQR) | 2 (0-3) | 3 (2-4) | P=0.001 |
